# The effect of LacI autoregulation on the performance of the lactose utilization system in *Escherichia coli*

**DOI:** 10.1093/nar/gkt351

**Published:** 2013-05-08

**Authors:** Szabolcs Semsey, Liselotte Jauffred, Zsolt Csiszovszki, János Erdőssy, Viktor Stéger, Sabine Hansen, Sandeep Krishna

**Affiliations:** ^1^Center for Models of Life, Niels Bohr Institute, University of Copenhagen, 2100 Copenhagen, Denmark, ^2^Laboratory of Molecular Biology, Center for Cancer Research, National Cancer Institute, National Institutes of Health, Bethesda, MD 20892-4264, USA, ^3^Department of Genetics, Eötvös Lóránd University, H-1117 Budapest, Hungary, ^4^Agricultural Biotechnology Center, Szent-Györgyi Albert u. 4, 2100 Gödöllő, Hungary and ^5^National Centre for Biological Sciences, Bangalore 560065, India

## Abstract

The lactose operon of *Escherichia coli* is a paradigm system for quantitative understanding of gene regulation in prokaryotes. Yet, none of the many mathematical models built so far to study the dynamics of this system considered the fact that the Lac repressor regulates its own transcription by forming a transcriptional roadblock at the *O3* operator site. Here we study the effect of autoregulation on intracellular LacI levels and also show that cAMP-CRP binding does not affect the efficiency of autoregulation. We built a mathematical model to study the role of LacI autoregulation in the lactose utilization system. Previously, it has been argued that negative autoregulation can significantly reduce noise as well as increase the speed of response. We show that the particular molecular mechanism, a transcriptional roadblock, used to achieve self-repression in the *lac* system does neither. Instead, LacI autoregulation balances two opposing states, one that allows quicker response to smaller pulses of external lactose, and the other that minimizes production costs in the absence of lactose.

## INTRODUCTION

**Figure 1.**
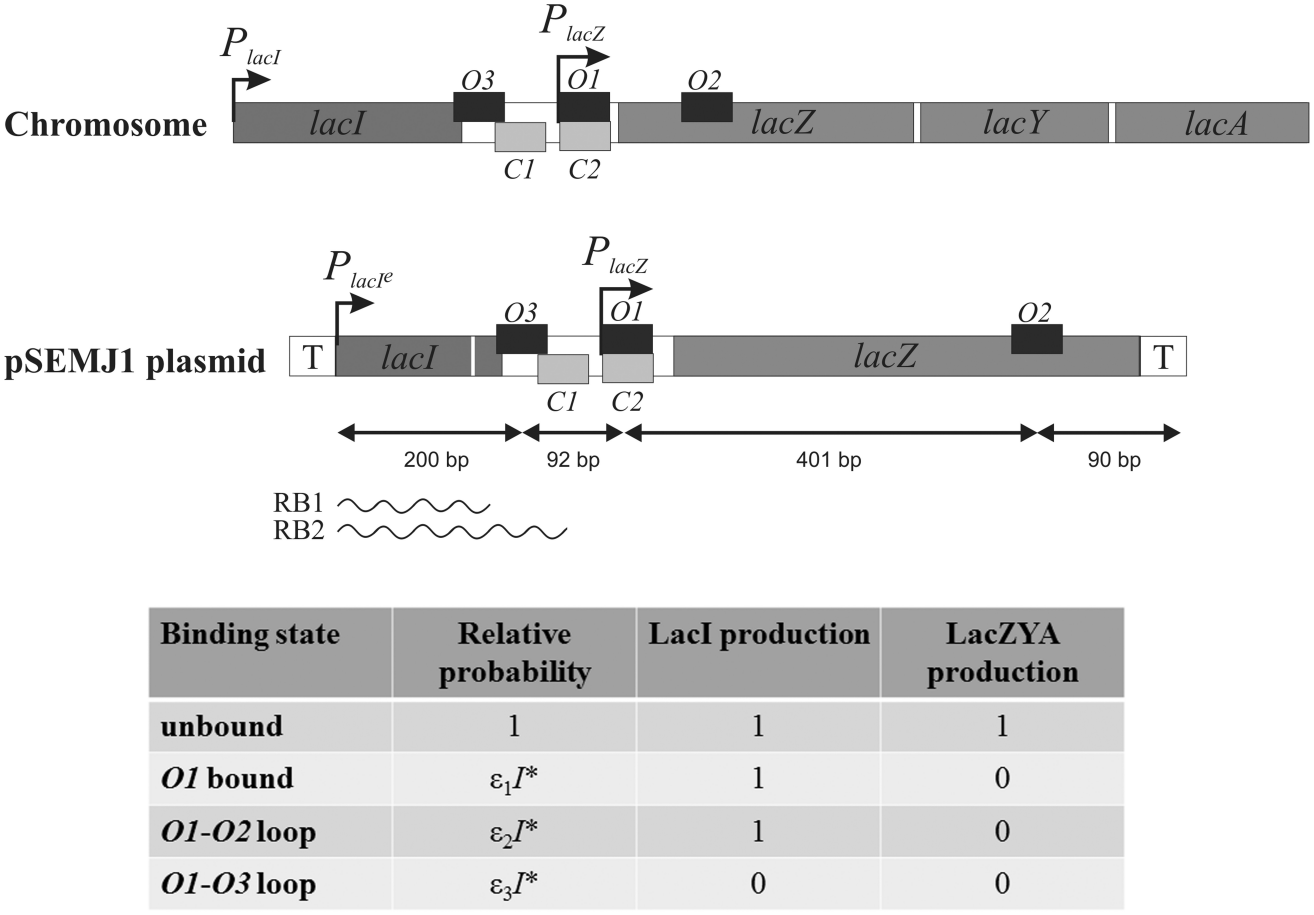
Schematic structure of the *lac* genes and control regions on the *E.coli* chromosome and in the pSEMJ1 plasmid, used as a template for *in vitro* transcription. Arrows represent promoters, smaller dark gray and light gray boxes represent LacI and cAMP-CRP binding sites, respectively. The lac region in the pSEMJ1 plasmid is flanked by transcription terminators (T). The *P_lacI_* - *O3* distance in pSEMJ1 is 1022 bp shorter than on the chromosome but the *O3-O2* region has the same sequence as the natural chromosomal gene. Transcripts initiated at *P_lacI_* can be roadblocked by LacI at *O3* (RB1) or at *O1* (RB2). The binding states considered in the model are shown in the bottom. Relative probabilities of the LacI-bound states depend on the active LacI concentration (I*) and on the associated binding energies (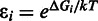, *ε_1_* ≈ 0.6 nM^−1^, *ε_2_* ≈ 13.8 nM^−1^, *ε_3_* ≈ 28.9 nM^−1^. The figure is not drawn to scale.

Bacteria sense a wide array of signals (minerals, nutrients, stress signals, etc.). A large class of cellular response systems regulates the flux and concentration of small molecules by controlling transport and metabolism pathways via two feedback loops connected by a common transcription regulatory protein that senses the intracellular concentration of the small molecule (1,2). In fact, almost half of the transcriptional regulators in *Escherichia coli* are directly regulated by a small molecule (3). The prototypic example of such a control system is the *lac* operon, which has been a paradigm of gene regulation. In *E. coli*, the *lac* operon contains genes encoding the lactose transporter (LacY) and the enzyme for lactose degradation (LacZ), therefore the lactose repressor (LacI) regulates the transport and metabolism pathways simultaneously (4). The *lacI* gene is present just upstream of the *lac* operon, and in fact there are three operator sites where the LacI tetramer can bind and affect transcription (5). The LacI tetramer contains two identical dimers, connected at their C-terminal region. Each dimer in the tetrameric structure has an N-terminal helix-turn-helix DNA-binding domain (6). The structure of the *lac* system is shown schematically in Figure 1. The main operator is *O1*, the strongest of the three operator sites. LacI binding to *O1* represses transcription of the *lac* operon but leaves the expression of the *lacI* gene unchanged. The binding of LacI to *O1* increases its probability to bind via DNA looping to *O2* or *O3*, which are weaker operators (7,8). When bound to *O1* and *O2*, transcription of the *lac* operon is repressed, while LacI continues to be produced. However, when *O1* and *O3* are bound, not only is the *lac* operon repressed, but the production of LacI is also prevented (9). In this state, transcription of *lacI* occurs but only a truncated transcript is produced, which is in turn subject to SsrA-mediated tagging and subsequent proteolysis of the truncated protein produced (9). While there is experimental evidence for LacI autoregulation (9,10), this feature of the network is ignored by the available mathematical models (11–15). Previous studies suggested that negative autoregulation in regulatory networks can significantly reduce noise (16) and increase the speed of response (17). In this work, we study the effect of autoregulation on intracellular LacI concentration and build a stochastic model of the lactose utilization system to explore the role of LacI autoregulation. We compare the natural *lac* system with two hypothetical controls, where LacI is produced at a constant low or at a constant high level, which correspond to the estimated autoregulated and fully expressed LacI levels, respectively. We show that the mechanism of LacI autoregulation neither reduces noise nor increases the speed of response. However, we find that the autoregulated system has a larger dynamic range and performs more economically than the constitutive systems.

## MATERIALS AND METHODS

### Plasmid construction

The pSEMJ1 plasmid, used as a template for *in vitro* transcription, was created by inserting the 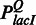 promoter region from plasmid pTYB1 (NEB) and the *P_lacZ_* promoter region (*O3-O2*, nt 365820 → 365101) from *E. coli* MG1655 (GeneBank: NC_000913.2) between the *Eco*RI and *Pst*I sites of plasmid pSEM2008. The 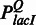 promoter region was polymerase chain reaction (PCR) amplified using the primers ATATATGAATTCGAATGTTGACAAACCTTTCGCGGTATGGCATGATAGC and ATATATCTCGAGATTCACCACCCTGAATTGACTCTCTTC to replace the original -35 promoter element with the TTGACA consensus sequence (the resulting enhanced promoter is termed 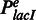). The *P_lacZ_* promoter region was amplified using the primers ATATATCTCGAGCAACTCTCTCAGGGCCAGGCGGTGAAGGGC and ATATATCTGCAG AATAATTCGCGTCTGGCCTTCCTGTAGCCAGC. The 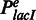 PCR fragment was cut with *Eco*RI and *Xho*I, and the *P_lacZ_* PCR fragment was cut with *Pst*I and *Xho*I. The two fragments were inserted between the *EcoR*I and *Pst*I sites of plasmid pSEM2008 by a three-piece ligation. The pSEM2008 plasmid was obtained by inserting the DNA fragment containing the *rrnBT1T2* terminators (nt 4559 → 4141) from pKK223-3 (Pharmacia, GeneBank M77749) between the *Kpn*I and *Eco*RI sites of pSA850 (18).

To create plasmid pSEM1068 for the expression of the His_6_-tagged dimeric LacI protein (lacking the last 16 amino acids), the *lacI* gene was PCR amplified using the primers AAAAGCTAGCAAAACCTTTCGCGGTATGGCTGAT and AAAAGAATTCAACGGAA GCACGTCGATCGGCCAAC, the amplified DNA fragment was digested with *Nhe*I and *Eco*RI, and inserted into the pSEM1026 vector (19) between the *Nhe*I and *EcoRI*I sites. The sequence of the amplified region was verified.

### Protein purification

The His_6_-tagged dimeric LacI protein was expressed in *E. coli* strain Top10 bearing pSEM1068 and purified using the protocol described previously for the purification of His_6_-tagged GalR (19). CRP was purified as described by Ryu *et al.* (20). The wild-type (WT) LacI protein was a kind gift from Maxim Sukhodolets.

### *In vitro* transcription and quantitation

**Figure 2.**
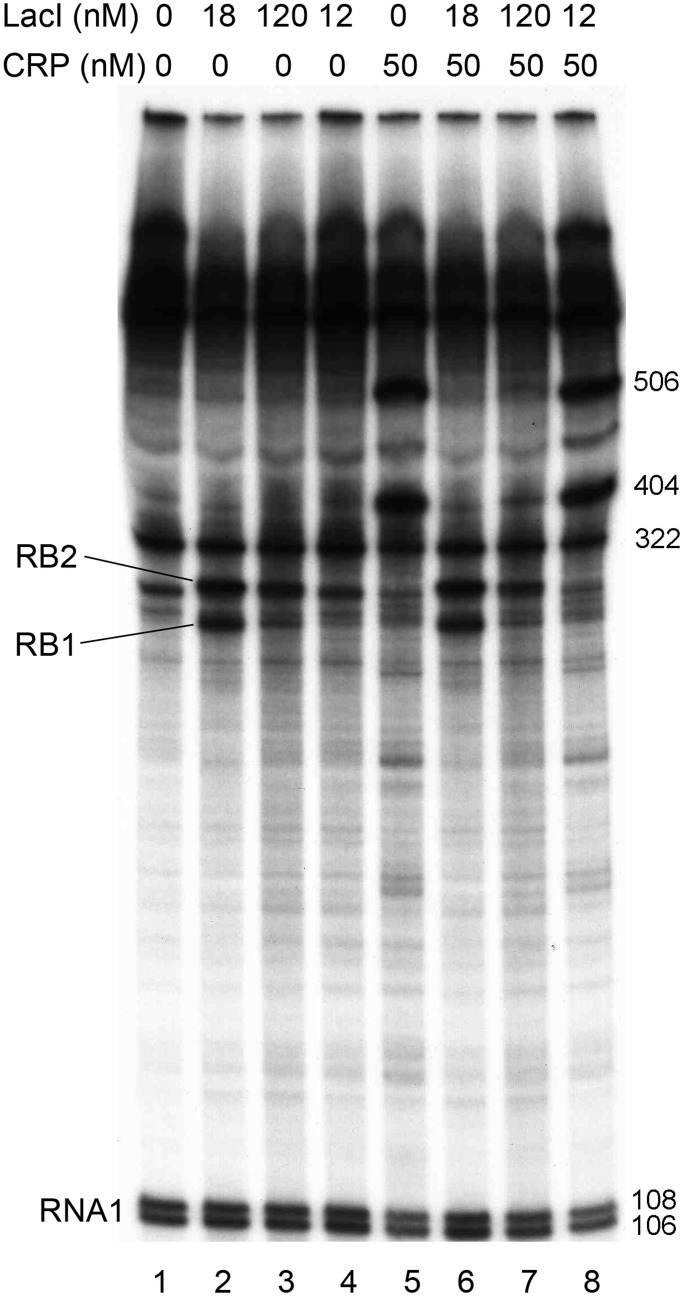
Effect of LacI and cAMP-CRP on transcription from the 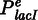 promoter *in vitro*. Concentrations of WT LacI (lanes 2 and 6), dimeric LacI (lanes 3, 4, 7 and 8) and CRP (lanes 5–8) are shown on top. cAMP was present at 100 µM concentration in all reactions. Two RNA species appeared in the presence of WT LacI as a result of roadblock termination (labeled RB1 and RB2). The RNA1 transcripts (106 and 108 nt) were used as internal controls between lanes. The lengths of RB1 and RB2 were estimated based on the migration distances of the RNA1 transcripts and of the transcripts initiated at promoters *P_lacZ_* (506 nt), *P_B_* (322 nt) and *P_C_* (404 nt) (36).

Reactions were performed on supercoiled pSEMJ1 plasmid DNA as described previously (21). LacI and CRP were used at the concentrations shown in Figure 2, when present. The RNA bands were quantified using the Storm 860 PhosphorImager (GE Healthcare). The lengths of the roadblocked transcripts were estimated based on the migration distances of transcripts with known lengths.

### Western blot and quantitation

Protein samples were loaded on a 10% Bis-Tris gel as follows: 10^10^, 5 × 10^10^, 10^11^, 2.5 × 10^11^ LacI repressor molecules mixed with cell extracts obtained from 2 × 10^8 ^*E. coli* MC4100 (Δ*lacI*) cells, extracts of 2 × 10^8^ MG1655 cells that were grown in the presence and absence of isopropyl β-d-thiogalactopyranoside (IPTG; 1 mM), respectively, and extracts obtained from 2 × 10^8 ^*E. coli* MC4100 cells.

After separation, the proteins were transferred to an Immobilon-P PVDF Membrane of 0.45 µm pore size (Millipore), then were blocked overnight with 5% nonfat dry milk in PBST (50 mM phosphate buffer at pH 7.4, 650 mM NaCl and 0.1% Tween 20). The blot was then incubated with primary antibody overnight at 4°C. It was then incubated with peroxidase-conjugated antibody and developed with Supersignal West Pico kit (Thermo Scientific). The dilutions of the antibodies were anti-LacI antibody (1:1000, Millipore), peroxidase-conjugated anti-mouse antibody (1:2000, Sigma A2554). Band intensities were quantified and background corrected. The bottom signal, which is also present in the *E. coli* MC4100 extract, was used as an internal control.

### Mathematical model

The dynamical variables we keep track of in our model are the concentrations of internal lactose (*L*), internal allolactose (*A*), LacI mRNA (*I_m_*), LacI tetramers (*I*), LacY permeases (*Y*) and the LacZ enzymes (*Z*). The deterministic differential equations that model the dynamics of these variables are described below:
(1)
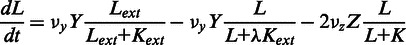

(2)
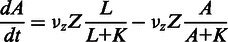

The first term on the right side of equation (1) represents the import of external lactose (*L_ext_*) by LacY, for which we have chosen a Michaelis–Menten form where *v_y_* is the maximum rate of import per LacY molecule, and the constant *K_ext_* is the *L_ext_* concentration at which the import rate per permease is half of its maximum value. The second term is similar and represents the export of internal lactose by LacY. The export has a different Michaelis constant, which is larger (*λK_ext_*). The final term models both hydrolysis of internal lactose as well as its conversion to allolactose, catalyzed by LacZ. Again a Michaelis–Menten form was chosen for these reactions, with each having a maximum rate of *v_z_* per LacZ. We further assume that both reactions have the same Michaelis constant K (22). The terms on the right side of equation (2) similarly model the production of allolactose and its hydrolysis by LacZ with the same Michaelis constant K (23). We assume that the binding and unbinding of allolactose to LacI is fast, so that we can take the concentration of active LacI (i.e. unbound to allolactose) to be
(3)
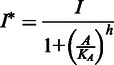

where *K_A_* is the Michaelis constant of allolactose-LacI binding and *h* is the associated Hill coefficient. Active LacI can bind to three operators *O1*, *O2* and *O3*. LacI tetramers bound to these operators can interact by the formation of DNA loops. If *O1* is bound, with or without a DNA loop, we assume that transcription of the *lac* operon is completely blocked. If *O3* is bound, we assume that transcription of *lacI* is roadblocked. In principle, there are then 14 possible states each with their particular combination of transcriptional repression of the *lacI* gene and the *lac* operon. In our model, we will only allow four states: (i) all operators are unbound, (ii) *O1* is bound, (iii) a DNA loop is formed between *O1* and *O2* and (iv) a DNA loop is formed between *O1* and *O3*. This is because the other bound states have significantly lower energies, and hence lower probability of occurrence, than the three bound states we allow (7). Promoter activity levels can be given as the sum of the products of promoter activities and probabilities for all possible states (24). Based on Figure 1, we can write the activities of the *lac* operon and the *lacI* gene as a function of the active LacI concentration (I*):
(4)
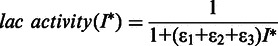

(5)
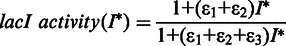

The *ε*s are related to the binding energies of the LacI-operator complexes, 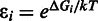. In addition, the activity of the *lac* operon is controlled by the cyclic-AMP-CRP level, *C*:
(6)
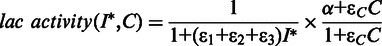

The effect of cAMP-CRP is taken to be completely independent of the effect of LacI on the total activity, as we show in Figure 2.

Using equations (5) and (6) we can write the differential equations for the relevant mRNA and protein levels:
(7)
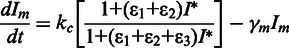

(8)
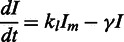

(9)
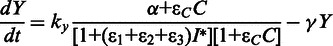

(10)
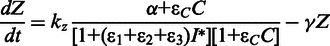

The *k*s are parameters that set the maximal rates of transcription and translation, and the *γ*s set the dilution and degradation rates of the proteins and *lacI* mRNA. We have chosen to model the *lacI* mRNA explicitly but not the *lac* operon mRNA because the former is produced at a sufficiently low rate (1 transcript per generation) (25,26) to produce significant fluctuations in the LacI levels, whereas the *lac* operon mRNA is produced at a high enough rate to have little effect on the fluctuations of LacY and LacZ levels.

For these deterministic differential equations, there is no need to have a separate equation for dZ/dt. Instead Z can simply be calculated from Y because 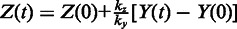. We simplify the model one step further by assuming that the transport of lactose and hydrolysis of lactose/allolactose take place much faster than the processes of transcription and translation. Then we can assume that *L* and *A* concentrations are always in quasi-equilibrium. Setting *dL*/*dt* = *dA*/*dt* = 0 makes the lactose and allolactose concentrations both equal to the physically sensible (i.e. real and non-negative) solution of the following:
(11)
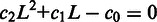

where
(12)
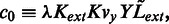

(13)
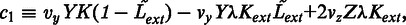

(14)
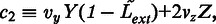

(15)
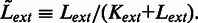

So,
(16)
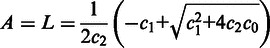

and the rest of the equations remain unchanged. Thus, equations 3, 7–10, and 12–16 are used for the deterministic simulations.

In the stochastic simulations, we keep track of the actual number of molecules of LacI tetramers, LacI mRNA, LacY and LacZ tetramers. The net production and degradation rates, from the equations of the deterministic model, expressed in appropriate units, can be treated as probabilities per unit time for the production and degradation of each species. We use the Gillespie algorithm (27) to determine, from these probabilities per unit time, the time at which the next production or degradation will happen and which species it will affect. We then accordingly increase or decrease the number of that species, recalculate the probabilities per unit time of production and degradation and repeat. This gives us a time series of the number of LacI tetramers, LacI mRNA, LacY and LacZ tetramer molecules as a function of time. Figures 5 and 6, and all statistics from them, were obtained from an ensemble of such stochastic simulations.

The number of parameters used in the model could be reduced by non-dimensionalizing the equations but this is not necessary here because we can fix most of the parameter values. Only the external lactose concentration (*L_ext_*) is varied in the simulations, the remaining parameters are always kept fixed.

### Parameter values:

The value of *γ* = 0.0087 min*^−^*^1 ^= ln(2)/(80 min) was chosen assuming a doubling time of 80 min (28) and no specific degradation of the proteins. The values used for the half-saturation constant for active transport by LacY (*K_ext_*) and the active transport turnover number (*v_y_*) were 0.27 mM and 48/s, respectively (29).

The *ε*s (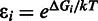) can be determined from the fold-repression of promoter activities in different conditions. In the WT cell with autoregulated level of LacI tetramers (30 nM), the repression of the *lac* operon activity is 1300-fold, and in a cell that has only the *O1* operator, the repression is 18-fold (7). Assuming that binding to the operator sites is sufficiently strong (to be precise, assuming 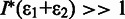), this sets *ε*_1_ ≈ 18/30 nM^−^^1^, and (*ε*_1_ + *ε*_2_ + *ε*_3_) ≈ 1300/30 nM^−^^1^. Further, the experimental results presented in this article set the ratio between the autoregulated and the fully expressed levels of LacI to be 1/3. Therefore *ε*_1_ + *ε*_2_ = *ε*_3_/2, i.e. *ε*_2_ = 415.33/30 nM*^−^*^1^ and *ε*_3_ = 866.66/30 nM*^−^*^1^. This choice results in 1300-fold repression in the deterministic simulations but a lower mean repression is observed in the stochastic simulations because of the noise in LacI levels. The repression level can be increased by increasing the LacI-operator binding energies or by increasing the number of lacI mRNAs produced per cell generation. We have tested these possibilities and the conclusions reported here were not affected.

We study our model only in conditions where the cAMP-CRP level is fixed and close to saturation, so we take the factor (*α* + *ε*_c_*C*)/(1 + *ε*_c_*C*) = 0.9.

For the allolactose–LacI interaction, we use a Hill coefficient of 2 and *K_A_* = 1 µM (12,30), while for LacZ-mediated hydrolysis of lactose and allolactose, we use *K* = 1.4 µM (22). The maximal transcription rate of the *lacI* mRNA (*k_c_*) is set to 1/80 nM/min because the *lacI* gene is transcribed approximately once per cell generation on average (25,26). The half-life of the *lacI* mRNA is ∼3.8 min (31), therefore *γ_m_* = ln 2/3.8 min = 0.1824/min. The maximal transcription rates of *lacY* (*k_y_*) and *lacZ* (*k_z_*) were chosen to be 90 and 100 nM/min, respectively, to obtain ∼10 µM LacY and slightly higher LacZ tetramer concentration when LacI is inactivated (32,33).

The combined conversion rate of lactose by LacZ (hydrolysis plus conversion to allolactose) is 3600/min (11), therefore *v_Z_* = 1800 min*^−^*^1^.

The maximal translation rate of the *lacI* mRNA (*k_l_*) was chosen to be 90 γγ_m_/*k_c_* per min to obtain 90 nM LacI tetramers/cell at saturating intracellular allolactose concentration.

This leaves one remaining parameter, λ, which sets the rate of LacY-mediated lactose export. To fix this, we need one additional constraint. We use the approximation that ∼3 billion glucose molecules are needed to generate a new cell (34). We found that with λ = 750 (dimensionless), with 5 mM external lactose concentration the cell, in our model, metabolizes ≈ 2 billion lactose molecules in 80 min, which must provide sufficient resources to generate a new cell.

## RESULTS

### *In vitro* transcription pattern of the lacI mRNA

*In vivo* studies of the distribution of different *lacI* mRNA species in rifampicin-treated cells showed that in the presence of LacI, transcription is blocked at the *O3* and *O1* operators with similar efficiencies (10). We constructed a plasmid DNA template that allows us to study how termination of *P_lacI_* transcription is controlled *in vitro*. The plasmid constructed (pSEMJ1) contained an enhanced *P_lacI_* promoter and the WT *lac* control region including both the *O3* and *O2* (Figure 1). The reason for using a control region spanning the whole *O3-O2* region instead of the 190-bp control region containing only *O3* and *O1* (10) was that a LacI tetramer can bind either *O1* and *O3* or *O1* and *O2*, and only the *O3-O1* loop can regulate *lacI* transcription. So far it is unclear how the choice between *O2* and *O3* binding is made by an *O1*-bound LacI tetramer. LacI bound to the *lac* control region efficiently separates the *lacI* and *lacZYA* transcription units; >90% of *lacI* transcription is blocked when both auxiliary operators are present (35). We performed *in vitro* transcription assays using supercoiled pSEMJ1 plasmid DNA to study the effect of LacI tetramers, LacI dimers and cAMP-CRP on the elongation of the *lacI* mRNA (Figure 2). In the presence of WT LacI, two RNA species appeared as a result of premature termination (labeled RB1 and RB2). However, in the presence of LacI dimers only a faint band appeared at the position of the shorter transcript (RB1), while the longer transcript (RB2) had similar intensity to the one obtained with WT LacI, suggesting that RB1 corresponds to a transcript terminated by LacI occupying *O3*, and RB2 is terminated owing to LacI binding to *O1*. Length estimation of the roadblocked transcripts suggest that RB1 and RB2 correspond to the *in vivo* observed mRNAs with endpoints III’ and II (10), respectively. The effect of cAMP-CRP on *O3-O1* loop formation is ambiguous in the literature (37,38). In our assays, the presence of both WT LacI and cAMP-CRP resulted in the same pattern obtained with WT LacI only (Figure 2, lane 2 versus lane 6). The amount of RB2 transcript obtained in the presence of LacI dimers was slightly reduced in the presence of cAMP-CRP (Figure 2, lanes 3 and 4 versus lanes 7 and 8). Therefore we concluded that even if cAMP-CRP is bound to the *O3-O1* loop, its presence does not affect the quantity of the RB1 and RB2 roadblock products, and it does not act as an additional roadblock. Therefore, in the mathematical model, the effect of cAMP-CRP is taken to be completely independent of the effect of LacI.

### The effect of autoregulation on *in vivo* LacI levels

**Figure 3.**
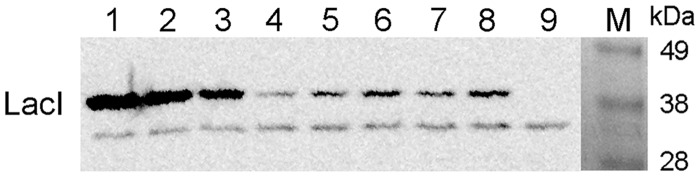
The effect of autoregulation on *in vivo* LacI levels. *Escherichia coli* MG1655 cells were grown in the absence (lanes 5 and 7) and in the presence (lanes 6 and 8) of 1 mM IPTG and proteins of 2 × 10^8^ cells were separated by sodium dodecyl sulphate-polyacrylamide gel electrophoresis and analyzed by western blotting, using an anti-LacI antibody. Purified LacI repressor molecules were mixed with proteins from 2 × 10^8^ cells of *E.coli* MC4100 (Δ*lacI*) (41) and loaded as controls (lanes 1–4, 2.5 × 10^11^, 10^11^, 5 × 10^10^, and 10^10^ LacI monomers, respectively. Proteins from 2 × 10^8^ cells of *E.coli* MC4100 were loaded in lane 9. The amount of LacI in cells was estimated by quantifying the signals obtained.

Most of the current models assume that cells contain ∼10 LacI tetramers, based on the estimate of Gilbert and Müller-Hill (39). A more recent work predicted 8.8 tetramers on average in HG104 (*ΔlacZYA*) cells as a lower bound on the actual number of proteins *in vivo* (40). We measured the average LacI content of cells in the presence and absence of IPTG, corresponding to fully expressed and autoregulated levels respectively, using western blotting of known amount of cells (Figure 3). Based on the experiment, we estimate that ∼150–180 monomers are present on average per cell in the presence of IPTG, while only 55–65 monomers/cell can be found in the absence of IPTG. Based on our estimate, the upper bound on the number of LacI tetramers is ∼40 molecules on average in the fully induced cells and 15 molecules in the absence of inducer. The latter falls between the estimate of 8.8 tetramers in HG104 cells and the theoretical calculation of 30 nM LacI tetramers by Santillan and Mackey (11).

### Development of the mathematical model for the lac system

We have developed a mathematical model to study the effects of LacI autoregulation. The model is described in detail in the ‘Materials and Methods’ section. Using this model, we compared the steady state and dynamic behaviors of the natural *lac* system, where LacI expression is autoregulated, with two hypothetical controls where LacI is produced at a constant low or at a constant high level, which correspond to the estimated autoregulated and fully expressed LacI levels, respectively. Because of the uncertainty of intracellular concentration of LacI tetramers, we have performed two sets of each computation, reflecting the higher and lower estimates found in the literature. In the first set, the average autoregulated LacI level was 30 nM and the fully expressed level was 90 nM, while in the second set these values were 10 and 30 nM, respectively.

### Steady-state simulations

**Figure 4.**
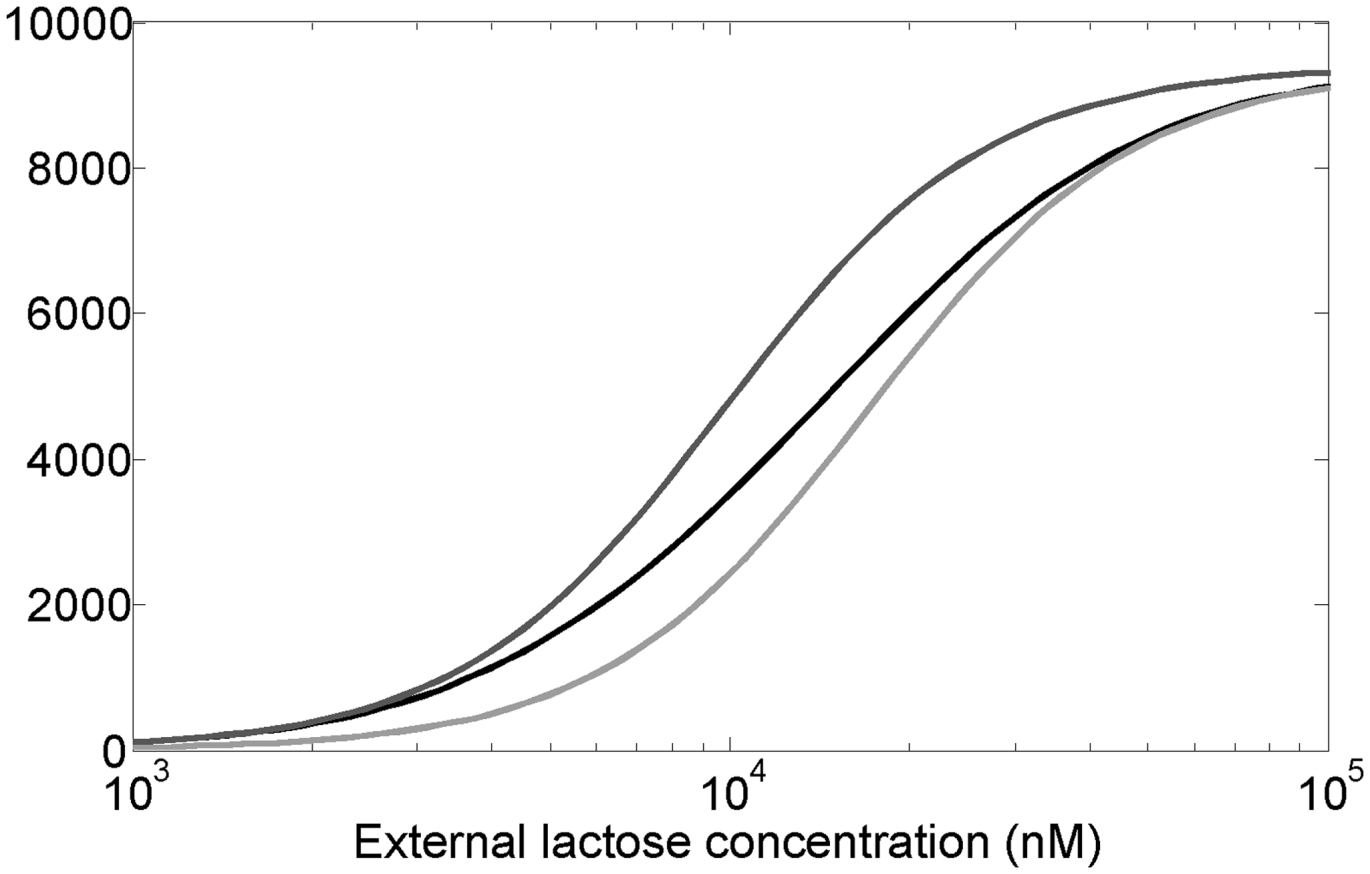
Average number of LacY molecules per cell as a function of extracellular lactose concentration, obtained by numerically solving the deterministic differential equations that define the model (see ‘Materials and Methods’ section). The black curve represents the WT system, while the dark gray and light gray curves represent the systems with non-regulated LacI expression, corresponding to 30 nM (fixed-low) and 90 nM (fixed-high) LacI levels, respectively. For large external lactose concentrations, the LacY number approaches ∼9400 molecules (we assume 1 nM is approximately one molecule per cell). Dynamic range (external lactose level at which 90% of this level is reached divided by external lactose level at which 10% of this level is reached) is ∼14.4 for WT, 9.2 for fixed-low and 9.4 for fixed-high systems.

In case of the *lac* system the input dynamic range can be defined as the extracellular lactose concentration (input) interval over which the average level of LacY transporter (output) changes significantly. Previously, systems controlled by negatively autoregulated regulators were found to have a larger input dynamic range and a more linear dose-response compared to similar systems regulated by constitutively expressed regulators (42,43). To explore the effect of LacI autoregulation on the input dynamic range of the system we have computed the average level of the LacY lactose transporter per cell, at different extracellular lactose levels, using deterministic simulations (Figure 4). In defining the input dynamic range as the ratio of extracellular lactose levels at which the system shows 90 and 10% of its maximal output, we follow Goldbeter and Koshland (44). The plot shows that the WT system has ∼50% larger dynamic range compared with the systems where LacI is present at constant low (30 nM) or high (90 nM) levels. A quantitatively similar increase in the dynamic range was obtained when LacI concentrations ranged from 10 to 30 nM instead.

**Figure 5.**
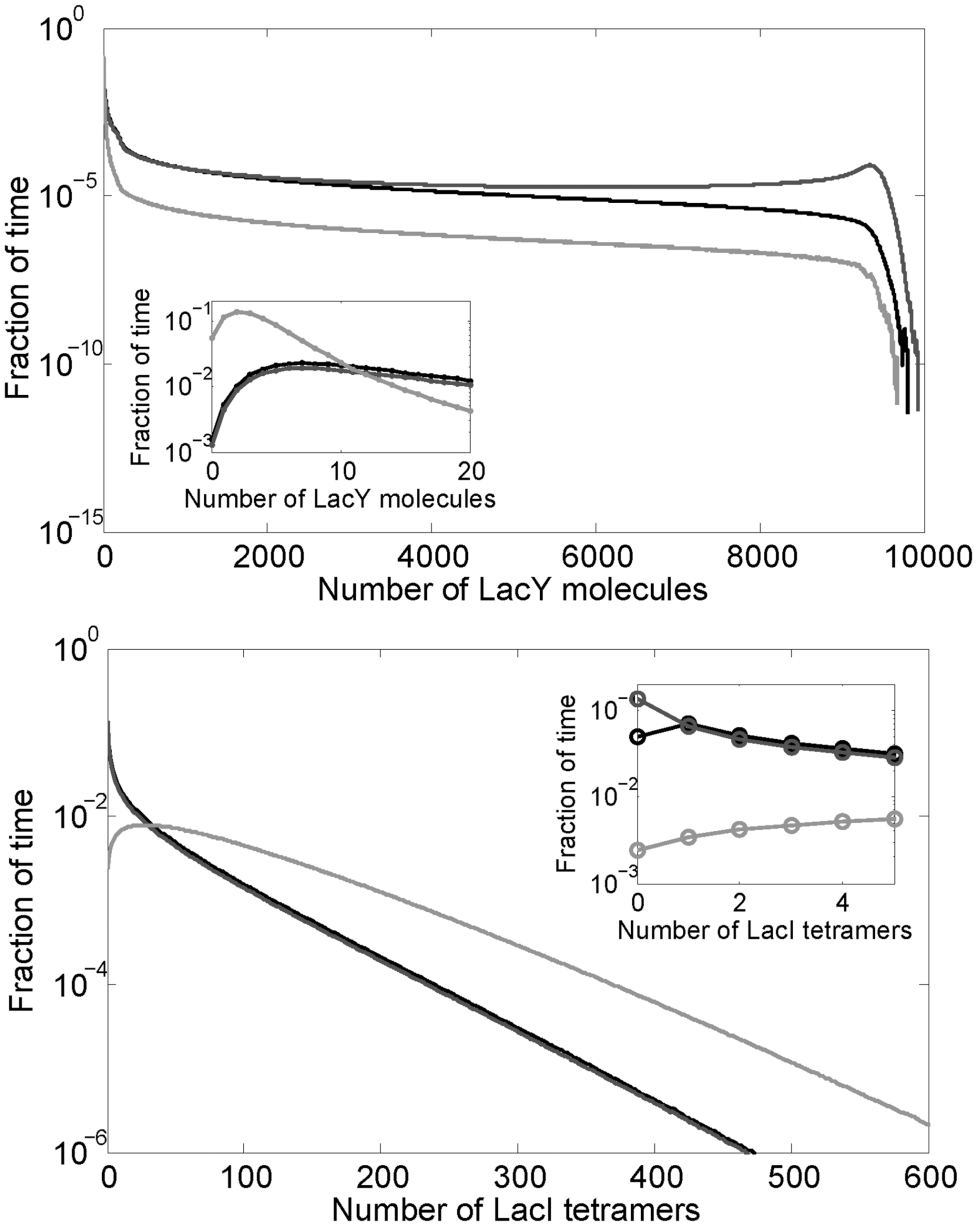
Distribution of the numbers of LacY (top) and LacI (bottom) molecules from stochastic simulations with zero external lactose (duration of 11 million min). The first 1 million min were discarded before making these distributions to eliminate any transients. The black curve represents the WT system, while the dark gray and light gray curves represent the fixed-low and fixed-high systems, respectively. Table 1 lists the mean and standard deviations for each distribution.

The *lacI* gene is transcribed from a weak promoter resulting in about one new *lacI* mRNA per cell generation (25,26). Due to the noise originating in stochastic intracellular processes and the low number of short-lived *lacI* mRNAs, repressor levels fluctuate with time within each cell and differ among isogenic cells (45). Negative autoregulatory feedback loops in gene circuits have been shown to limit the range over which the concentrations of network components fluctuate (16). We performed stochastic simulations at zero extracellular lactose concentration to compare LacI and LacY distributions in the constitutive and autoregulated systems (Figure 5). Interestingly, similar LacI levels were found in the WT system (mean ± SD = 33.1 ± 43.7) and in the system having constant low level of LacI (30 ± 42.7). Thus, autoregulation does not seem to reduce noise in LacI levels compared with the constitutive low system. Nevertheless, the LacY level in the model is much higher for the system where LacI is expressed at a constant low level (1306 ± 2566) compared with the WT system (491.8 ± 1271). This is because probability of having zero intracellular LacI was about three times lower in the WT system (Figure 5 inset), which influences the average LacY levels significantly. We also computed the protein levels for the system where LacI is expressed at a constant high level (90 nM). Because of its higher level, the intracellular LacI level fluctuates less (90 ± 74 nM), and results in a more successful repression of the *lac* operon. As a consequence of stronger repression, the LacY level was lower and the variability of the LacY level was found to be substantially higher (29.9 ± 301.6 nM). Similar results were obtained in a second set of simulations where the LacI levels were in the range of 10–30 nM (Table 1).

**Table 1.** Mean ± standard deviations of the numbers of LacI and LacY molecules from stochastic simulations, with zero external lactose (Figure 5)

	LacI range: 30–90 molecules			LacI range: 10–30 molecules		
	WT (autoregulated)	Constant low	Constant high	WT (autoregulated)	Constant low	Constant high
LacI molecules	33.1 ± 43.7	30.0 ± 42.7	90.0 ± 74.0	11.9 ± 15.1	10.1 ± 14.6	30.1 ± 25.2
LacY molecules	491.8 ± 1271	1306 ± 2566	29.9 ± 301.6	851.7 ± 1658	2119 ± 3065	113.4 ± 651.2

The last three columns are for simulations where the rate of translation of LacI mRNA is one-third of the default value described in ‘Materials and Methods’ section, thereby LacI levels range from 10 to 30 tetramers (the binding energies were appropriately modified to have the same repression levels).

### Dynamic simulations

**Figure 6.**
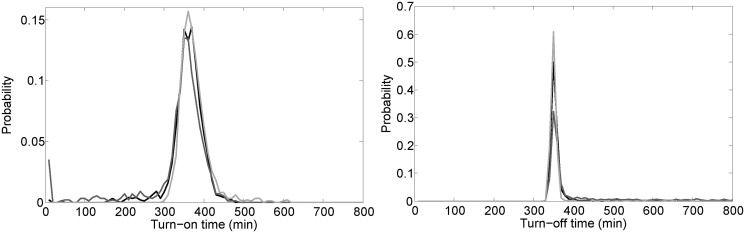
Distributions of turn-on time (left) and turn-off time (right) obtained from 1000 stochastic simulations where the external lactose concentration was zero for t = 0 min to t = 10 000 min, then external lactose was fixed at 5 mM for t = 10 000 min to t = 20 000 min, and finally external lactose was again set to zero from t = 20 000 min to t = 30 000 min. The long times between changes of external lactose were chosen simply to allow enough time for the system to reach steady state before each change. Turn-on time for each simulation was the time after t = 10 000 min required for the LacY level to first reach 9025 molecules. The black curve represents the WT system, while the dark gray and light gray curves represent the fixed-low and fixed-high systems, respectively. Turn-off time for each simulation was the time after t = 20 000 min required for the LacY level to first reach 475 molecules. Table 2 lists the mean and standard deviation for each distribution.

*Escherichia coli* cells need to optimize their gene expression pattern in environments where the quality and amount of carbon sources fluctuate, most likely in an unpredictable fashion (46). To test how fast the WT system responds to changes in extracellular lactose levels compared with the systems having constitutive LacI expression, we performed stochastic simulations where the external lactose level was changed from 0 to 5 mM, and later back to 0 nM. We recorded the turn-on and turn-off times, which are defined as the time taken to reach 95 and 5% of the maximal LacY levels, respectively (Figure 6). We find that the system expressing LacI constitutively at a low level has a longer turn-off time on average, and higher population heterogeneity in both turning on and off (Table 2). Furthermore, although the average turn-on times are similar, the system with fixed high LacI almost always takes much more than a cell generation to turn on, whereas some cells in the WT and fixed-low systems turn on at times even much less than a cell generation. More precisely, at 299 min after the concentration of external lactose jumped from 0 to 5 mM, we found that 0 out of 1000 cells with fixed high LacI had turned on, whereas 52 and 139 cells out of 1000 had turned on in the WT and fixed low systems, respectively. Again, similar results were obtained in the second set of computations where LacI ranges from 10 to 30 nM.

**Table 2.** Mean ± standard deviations of turn-on and turn-off times obtained in the simulations shown in Figure 6

	LacI range: 30–90 molecules			LacI range: 10–30 molecules		
	WT (autoregulated)	Constant low	Constant high	WT (autoregulated)	Constant low	Constant high
Turn-on time (minutes)	354.2 ± 41.7	330.5 ± 85.4	368.8 ± 36.0	349.3 ± 44.2	308.7 ± 103.7	364.8 ± 38.3
Turn-off time (minutes)	363.7 ± 74.4	468.9 ± 246.0	348.4 ± 33.0	394.0 ± 128.6	602.0 ± 392.5	354.4 ± 50.5

The last three columns are for simulations where the rate of translation of LacI mRNA is one-third of the default value described in ‘Materials and Methods’ section, thereby LacI levels range from 10 to 30 tetramers (the binding energies were appropriately modified to have the same repression levels).

## DISCUSSION

Autoregulation is a common feature of sugar-specific transcription regulatory proteins in *E. coli* (47). Negative autoregulation typically reduces the rate of transcription initiation (48–50), however, in the lactose system of *E. coli* transcription elongation is inhibited (9). The *lac* system is intrinsically noisy because of the low probability of *lacI* transcription (26) and because of the topology of the regulatory elements, i.e. simultaneous transcriptional regulation of both the *lacI* gene and the *lac*ZYA operon by a single LacI tetramer bound to *O1* and *O3*. The intrinsic noise can generate heterogeneous expression of LacY and LacZ in the cells of *E. coli* populations in the absence of lactose (45). The *lac* system was also found to exhibit bistable behavior in the presence of low levels of non-metabolized inducers such as thiomethyl β-d-galactoside and IPTG but no bistability was observed when lactose was used as an inducer (14,51).

The capability of negative transcriptional autoregulation to reduce gene expression noise has been demonstrated both experimentally and theoretically (16,52). However, strong negative autoregulation was also reported to have the opposite effect, an increase in the protein variability sacrificed for reduced mRNA usage (53). Cell to cell heterogeneity can be either beneficial or disadvantageous, and therefore regulatory systems may evolve either to reduce or maintain it. We have addressed the effect of negative autoregulation of LacI on protein level variability in the absence of inducer theoretically, by building a mathematical model and performing stochastic simulations. Results of simulations suggest that the noise in LacI expression in the absence of inducer, quantiﬁed as the squared coefﬁcient of variation (54), is not smaller in the WT (negatively autoregulated) system than in the constitutive systems expressing constant low or high levels of LacI. Interestingly, even though the mean LacI level and noise is similar in the negatively autoregulated and constitutive low systems, the mean LacY level was found to be about two times higher in the constitutive system. This is because it is more probable to have zero LacI in a cell in the constitutive system. Cells having zero LacI express genes of the *lac* operon at a high level but can use lactose immediately when it becomes available. Negative autoregulation of LacI decreases the probability of having zero LacI in the cell, from which we can speculate that in natural habitats the cost associated with higher LacY expression and slower turn off of the constitutive low system is higher than the potential benefit of fast lactose utilization in a fraction of the population. Unlike the constitutive low system, the constitutive high system is at least as economical as the WT system in the absence of lactose—although it expresses LacI at a higher level, expression of the *lac* operon is much lower in this system. However, cells with constitutive high LacI levels would perform worse than constitutive low or WT cells in an environment where these systems compete for small pools of lactose that appear rarely and intermittently. This is because a fraction of the populations having the constitutive low or WT systems can start to utilize the lactose source much earlier than the average turn-on time, while the constitutive high system lacks this opportunity.

Because the LacI level can be changed in the WT system, this system balances the two opposing states, one that allows quicker response to smaller pulses of external lactose, and the other that minimizes production costs in the absence of lactose. The resulting increased dynamic response range therefore enhances the overall performance of the autoregulated *lac* system.

## FUNDING

Danish Council for Independent Research|Natural Sciences; the Intramural Research Program of the National Institutes of Health; National Cancer Institute; Center for Cancer Research and the Danish National Research Foundation. Funding for open access charge: Danish National Research Foundation.

*Conflict of interest statement.* None declared.
